# *CEP55*-associated lethal fetal syndrome: a case report of a Chinese family

**DOI:** 10.3389/fgene.2023.1267241

**Published:** 2023-10-20

**Authors:** Yeping Wang, Fang Sheng, Lingjing Ying, Qiaoli Lou, Zhaonan Yu, Kaixuan Wang, Haoyi Wang

**Affiliations:** ^1^ Jinhua Maternity and Child Health Care Hospital, Jinhua, China; ^2^ Jinhua Municipal Central Hospital, Jinhua, China; ^3^ Wuyi County First People’s Hospital, Jinhua, China; ^4^ Medical School of Tianjin University, Tianjin, China; ^5^ Hangzhou D. A. Medical Laboratory, Hangzhou, China; ^6^ Precision Diagnosis and Treatment Center of Jinhua City, Jinhua, China

**Keywords:** *CEP55*, stillbirth, Meckel syndrome, case report, fetal loss

## Abstract

**Background:** Research on fetal loss related to germline mutations in single genes remains limited. Disruption of *CEP55* has recently been established in association with perinatal deaths characterized by hydranencephaly, renal dysplasia, oligohydramnios, and characteristic dysmorphisms. We herein present a Chinese family with recurrent fetal losses due to compound heterozygous nonsense *CEP55* variants.

**Case presentations:** The Chinese couple had a history of five pregnancies, with four of them proceeding abnormally. Two stillbirths (II:3 and II:4) sequentially occurred in the third and fourth pregnancy. Prenatal ultrasound scans revealed phenotypic similarities between fetuses II:3 and II:4, including oligohydramnios, bilateral renal dysplasia and hydrocephalus/hydranencephaly. Clubfoot and syndactyly were also present in both stillborn babies. Fetus II:3 presented with endocardial cushion defects while fetus II:4 did not. With the product of conception in the fourth pregnancy, whole exome sequencing (WES) on fetus II:4 identified compound heterozygous nonsense *CEP55* variants comprised of c.190C>T(p.Arg64*) and c.208A>T(p.Lys70*). Both variants were expected to result in lack of the TSG101 and ALIX binding domain. Sanger sequencing confirmed the presence and cosegregation of both variants.

**Conclusion:** This is the fifth reported family wherein biallelic *CEP55* variants lead to multiple perinatal deaths. Our findings, taken together with previously described phenotypically similar cases and even those with a milder and viable phenotype, broaden the genotypic and phenotypic spectrum of *CEP55*-associated lethal fetal syndrome, highlighting the vital biomolecular function of CEP55.

## Introduction

Fetal loss, one of the most severe adverse outcomes of pregnancy, can lead to long-lasting grief, guilt, anxiety, self-blame, post-traumatic stress disorder, and marriage breakdown (Robinson, 2014; Smith et al., 2022). The annual prevalence of fetal loss at ≥20 weeks (stillbirth) is estimated at 13.2 and 5.74 per 1,000 births in China and the United States, respectively (Zhu et al., 2021; Gregory et al., 2022). A subgroup of fetal losses have been attributed to Mendelian diseases caused by pathogenic single-nucleotide variants or small insertions/deletions, though the relevant data are limited (Shamseldin et al., 2018; Sahlin et al., 2019; Stanley et al., 2020).

The *Centrosomal Protein 55 kDa* (*CEP55*) gene located at chromosome 10q23 encodes a coiled-coil centrosomal protein that promotes the abscission process, the second stage of cytokinesis, through the recruitment of Endosomal Sorting Complex Required for Transport (ESCRT) machinery to the midbody (Fabbro et al., 2005; Lee et al., 2008). This protein harbors 464 amino acids with three central coiled-coil domains, and phosphorylation of the three C-terminal serine residues (p.S425, p.S428, p.S436) is indispensable to abscission (Fabbro et al., 2005; van der Horst et al., 2009). Recently homozygous truncating mutations in *CEP55* have been identified as a cause of a lethal fetal condition, termed Meckel-like syndrome or MARCH syndrome (acronym for Multinucleated neurons, Anhydramnios, Renal dysplasia, Cerebellar hypoplasia, and Hydranencephaly, OMIM #236500), characterized by fetal loss with severe congenital malformations including hydranencephaly, renal dysplasia, oligohydramnios, and characteristic dysmorphisms (Bondeson et al., 2017; Frosk et al., 2017). To date only three studies have delineated this lethal *CEP55*-associated syndrome, though viable phenotypes linked to biallelic *CEP55* variants have also been reported (Bondeson et al., 2017; Frosk et al., 2017; Rawlins et al., 2019; Barrie et al., 2020).

We herein present a Chinese family with a history of recurrent fetal loss, in which multiple fetuses present with complex brain and kidney malformations, oligohydramnios, and physical anomalies. Whole exome sequencing (WES) reveals compound heterozygous variants comprised of two nonsense mutations in the *CEP55* gene, NM_018131.4(*CEP55*): c.190C > T(p.Arg64*) and c.208A > T(p.Lys70*). Given that the relevant studies are scarce, our case report expands the genotypic and phenotypic spectrum, thereby providing a better understanding of *CEP55*-associated lethal fetal syndrome.

## Case presentation

### Clinical report

**FIGURE 1:**
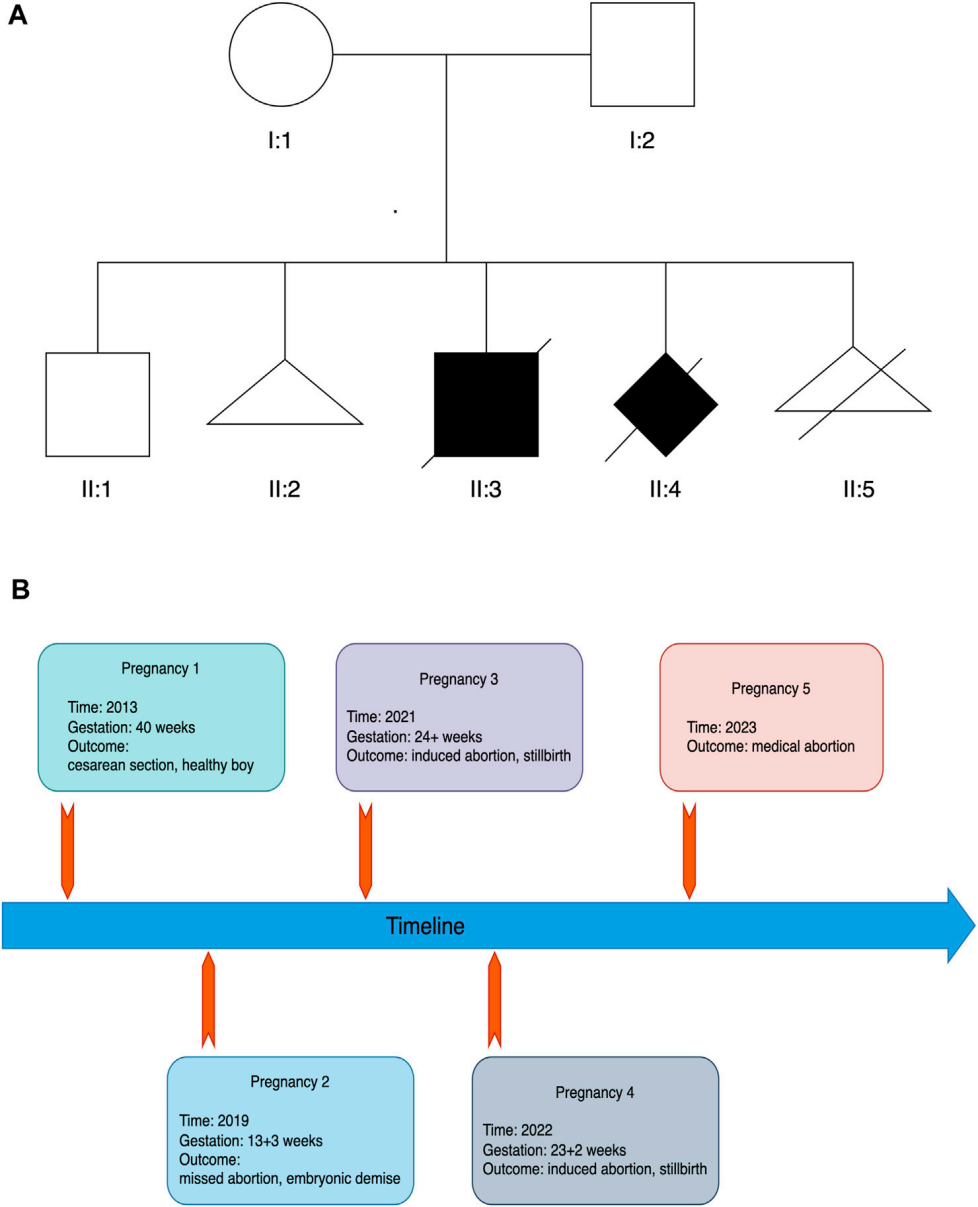
Pedigree **(A)** and timeline of pregnancies **(B)** in the family.

The Chinese couple with five past pregnancies presented to our clinic with a history of recurrent pregnancy loss (Figure 1). In accordance to patient inquiries, they had no known consanguinity and history of antepartum teratogenic exposures. In the first pregnancy, nothing abnormal but oligohydramnios was documented and a healthy boy (II:1) was born by cesarean section in 2013. This 11-year-old boy has no any congenital defect or serious disease to date. The second pregnancy ended by a missed abortion due to embryonic demise in gestational week (GW) 13 + 3 in 2019 (II:2). No clinical or genetic material was available.

**FIGURE 2:**
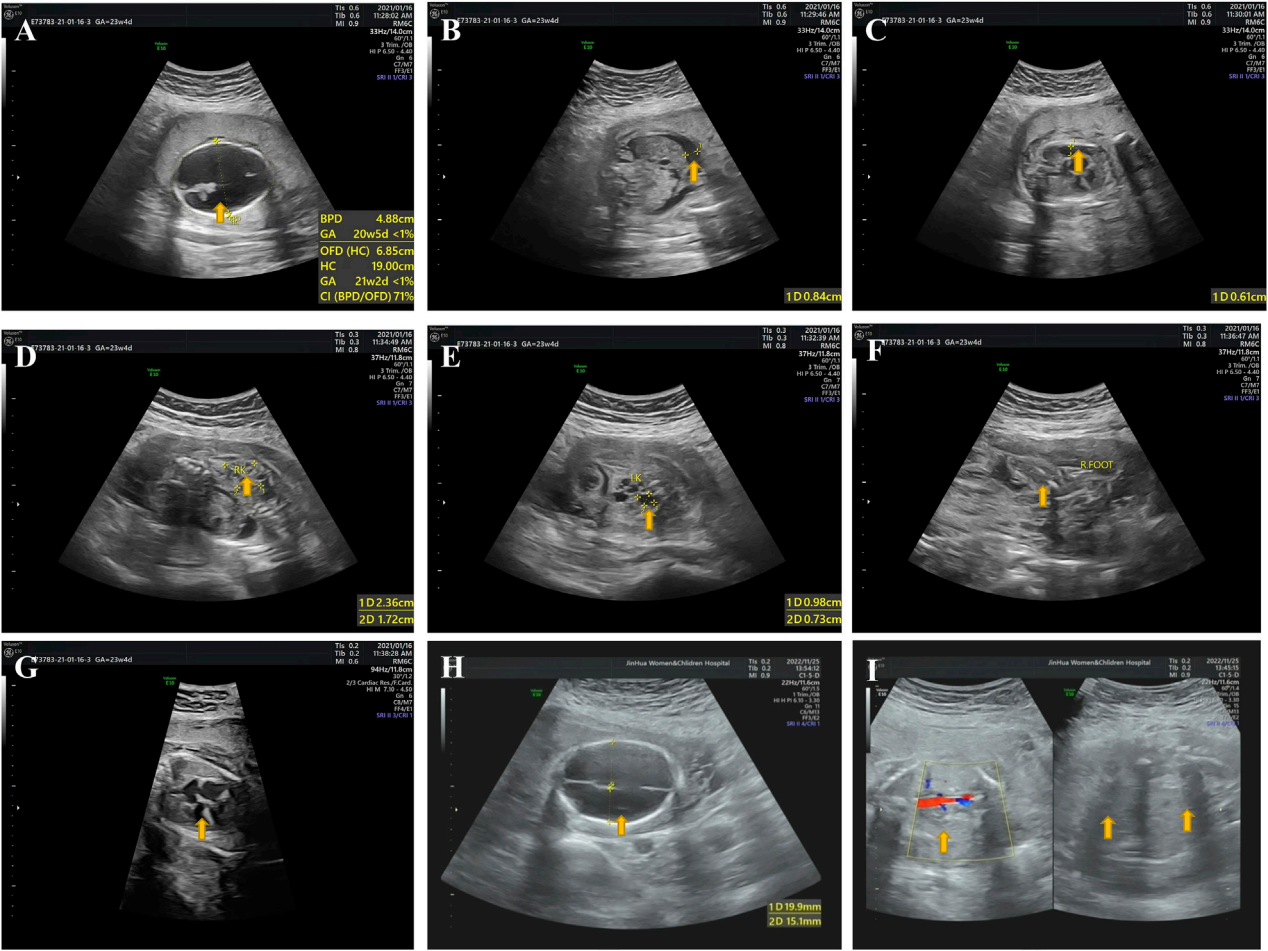
Ultrasound pictures from the third and fourth pregnancy (fetuses II:3 and II:4). **(A)** II:3: hydrocephalus. **(B)** II:3: ascites. **(C)** II:3: pleural effusion. **(D)** II:3: polycystic dysplasia of right kidney. **(E)** II:3: polycystic dysplasia of left kidney. **(F)** II:3: clubbed right foot. **(G)** II:3: endocardial cushion defects. **(H)** II:4: hydranencephaly. **(I)** II:4: bilateral renal agenesis. Yellow arrows indicate where the anomalies occur.

In the third pregnancy, a first trimester ultrasound scan (GW 12 + 2) revealed soft tissue swelling throughout the fetus (II:3) with an increased thickness of nuchal translucency (2.7 mm). A second trimester ultrasound scan was then performed at GW 21 + 4 and showed that fetus II:3 presented with hydrops, severe hydrocephalus (Figure 2A), ascites (Figure 2B), pleural effusion (Figure 2C), oligohydramnios, polycystic dysplasia of bilateral kidneys (Figures 2D, E), clubfoot (Figure 2F) and endocardial cushion defects (Figure 2G). Of note, a clear view of detailed brain structures by ultrasound scan was unavailable due to the fetal position, and thus we could not rule out the possibility of hydranencephaly. Owing to the fetal anomalies and the predicted lethal outcome, the pregnancy was terminated by an induced abortion at GW 24+ in 2021. A stillborn male fetus weighing 680g was delivered, displaying neck swelling, generalized edema, clubbed feet and syndactyly.

In the fourth pregnancy, a second trimester ultrasound scan (GW 23 + 1) revealed a fetus (II:4) with hydranencephaly (fluid-filled sacs and missing cerebral tissues, preservation of cerebral midline and thalamus, non-visualized cerebellum and posterior fossa structures; Figure 2H), anhydramnios (amniotic fluid index: 0 cm) and bilateral renal agenesis (Figure 2I). Face and limbs could not be identified. Also, ultrasound measurements indicated a fetal age at GW 20 + 1, suggesting an intrauterine fetal growth restriction (IUGR). Like the third pregnancy, this pregnancy ended by an induced abortion at GW 23 + 2 in 2022, leading to a stillbirth of a fetus of unknown gender manifesting syndactyly and clubfoot. Postmortem histological examinations were not performed on fetuses II:3 and II:4 owing to severe autolysis.

The fifth pregnancy accidentally occurred in 2023. After a genetic consultation, the couple voluntarily abandoned this pregnancy and a medical abortion was thus administered.

### Genetic analysis

**FIGURE 3:**
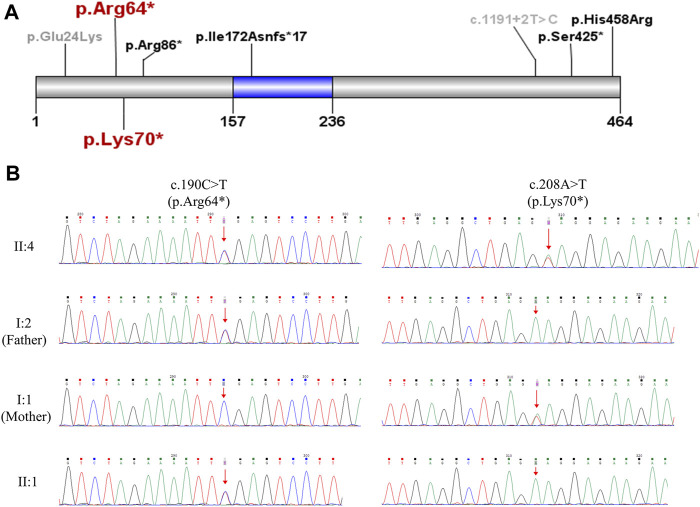
Genetic analysis of the *CEP55* variants. **(A)** Schematic representation of CEP55 protein indicating the position of the disease-associated variants. The region marked in blue (residue 157–236) is tumour suppressor gene 101 (TSG101) and apoptosis-linked gene 2 interacting protein X (ALIX) binding domain. The two variants p.Arg64* and p.Lys70* identified in the current study are highlighted in red. The variants that caused perinatal deaths in previous studies (Bondeson et al., 2017; Frosk et al., 2017; Rawlins et al., 2019; Sparks et al., 2020) are marked in black. The variants identified in viable cases (Barrie et al., 2020) are marked in gray. **(B)** Sanger sequencing validation of the two *CEP55* variants in the family.

To uncover genetic factors contributing to the fetal malformations, chromosomal microarray and WES were performed on fetus II:4 with the product of conception (POC). The chromosomal microarray analysis revealed no genetic abnormalities, excluding the possibility of pathogenic copy number variations. The WES analysis identified compound heterozygous nonsense mutations, NM_018131.4(*CEP55*): c.190C > T(p.Arg64*) and c.208A > T(p.Lys70*), both of which were predicted to result in a premature stop and produce a truncated CEP55 protein lacking the TSG101 and ALIX binding domain (Figure 3A). The nonsense *CEP55* variant p.Arg64* (ClinVar ID: 1065435) is classified as likely pathogenic in the ClinVar database. This variant in the compound heterozygous state has previously been reported in a case of nonimmune hydrops fetalis (NIHF) with a second missense *CEP55* variant p.His458Arg (Sparks et al., 2020). The nonsense *CEP55* variant p.Arg70* is not included in the ClinVar or HGMD databases. Both variants are listed in gnomeAD, but the lack of homozygotes and extremely low allele frequencies (1.208e−5 for p.Arg64* and 1.595e−5 for p.Lys70*) do not argue against causality for an autosomal recessive disease. Moreover, Sanger sequencing confirmed that both variants were located in trans and segregated with disease in the family (Figure 3B). According to the ACMG guidelines (Richards et al., 2015), we have classified these two variants as pathogenic. No other pathogenic variants were identified. Based on the clinical and genetic findings, fetus II:4 has been diagnosed with MARCH syndrome (or Meckel-like syndrome).

## Discussion

To our knowledge, this is the fifth described family with perinatal deaths caused by biallelic *CEP55* mutations (Table 1). It is with regret that we did not obtain materials to perform WES on fetus II:3 to consolidate our results because the third pregnancy was not followed up in our hospital. However, we believe that fetuses II:3 and II:4 harbor the same genetic aetiology given their high phenotypical similarities, including features of renal dysplasia, oligohydramnios, clubfoot, and central nervous system (CNS) abnormalities.

**TABLE 1:** Comparison of perinatal deaths due to biallelic *CEP55* variants.

References	Bondeson et al. (2017)		Frosk et al. (2017)			Rawlins et al. (2019)		Sparks et al. (2020)	This study	
Patient	II:2	II:3	303	305	306	VI:1	VI:3	H107	II:3	II:4
*CEP55* (NM_018131.4) genotype	c.256C > T p.Arg86*		c.1274C > A p.S425*			c.514dup p.Ile172Asnfs*17		c.190C > T p.Arg64* & c.1373A > G p.His458Arg	nk	c.190C > T p.Arg64* and c.208A > T p.Lys70*
Zygosity	hom		hom			hom		CH	nk	CH
Gender	M	M	M	M	M	M	F	nk	M	nk
Pregnancy outcome (gestation)	TOP (20)	IUFD (14 + 6)	SB (30)	ND (35)	SB (32)	SB (42)	ND (42)	SB (na)	SB (24)	SB (23)
Gestation when anomalies were identified	19 + 4	10 + 4	20	nk	nk	19	21 + 2	na	21 + 4	23 + 1
Renal features										
Renal aplasia/dysplasia	+	nk	+	+	+	nk	+	+	+	+
Renal cysts	+	nk	−	−	−	nk	+	−	+	−
Oligohydramnios	+	na	+	+	+	+	+	+	+	+
Clubfoot	+	na	+	+	+	+	+	+	+	+
CNS features										
Hydranencephaly	+	−	+	+	+	+	+	−	−	+
Cerebellar hypoplasia	+	nk	+	+	+	nk	nk	nk	nk	+
Multinucleated neurons	nk	nk	+	+	+	nk	nk	nk	nk	nk
Growth										
IUGR	+	nk	na	na	na	+	+	+	nk	+
Cardiac features										
Ventricular/atrial septal defect	nk	nk	nk	nk	nk	nk	nk	+	+	−
Aorta involvement	nk	nk	+	nk	+	nk	nk	+	nk	nk
Other features										
Cystic hygroma	+	+	+	+	+	−	−	+	nk	nk
Syndactyly	na	na	+	+	+	+	+	nk	+	+
Single umbilical artery	+	na	−	−	−	−	+	−	−	−
Redundant neck skin	na	na	+	+	+	+	+	−	+	−
Skin edema	nk	nk	nk	nk	nk	nk	nk	+	+	−

“+”, presence of a feature; “−”, absence of a feature; nk, not known; na, not available; hom, homozygous; CH, compound heterozygous; TOP, termination of pregnancy; IUFD, intrauterine fetal death; SB, stillbirth; ND, neonatal death; CNS, central nervous system.

The *CEP55*-associated lethal fetal syndrome was firstly defined by Bondeson et al. (2017) and Frosk et al. (2017) in 2017. Bondeson et al. (2017) termed this syndromic phenotype Meckel-like syndrome because phenotypic overlap with Meckel syndrome (MKS) was observed in *CEP55* fetuses. MKS is a lethal autosomal recessive ciliopathy characterized by a classic triad of renal cystic dysplasia, CNS anomalies and polydactyly. Of note, polydactyly was absent in all *CEP55* fetuses. With regard to CNS abnormality, encephalocele dominates MKS cases while hydranencephaly is present in most of *CEP55* fetuses (Barisic et al., 2015). In addition, hepatic ductal plate malformation is also a frequent feature of MKS on postmortem histological examinations. To date only three *CEP55* fetuses have been autopsied, none of whom displays this anomaly (Frosk et al., 2017). These phenotypic disparities may result from pathomolecular differences. In our view, the *CEP55*-associated lethal fetal condition could be considered a relatively severe form of MKS given the high incidence of hydranencephaly and renal agenesis. Indeed, *CEP55*-knockout mouse mutants exhibited microcephaly instead of hydranencephaly (Tedeschi et al., 2020; Little et al., 2021; Zhang et al., 2021), possibly due to interspecies differences concerning brain development between human and mouse.

The work by Frosk et al. (2017) complemented the anatomical and histological characteristics and potential pathogenesis of *CEP55*-associated lethal fetal syndrome via autopsy and *in vitro* and *in vivo* experiments. In addition to the phenotypes mentioned above, the three siblings described by Frosk et al. (2017) with a homozygous downstream nonsense mutation c.1274C > A (p.S425*) displayed multinucleated neurons on autopsy, leading the authors to term this condition MARCH syndrome. This cerebral histological abnormality resulted from abscission failure due to defective localisation of truncated protein to the midbody caused by *CEP55* downstream truncating variants (Frosk et al., 2017). Compared with nonsense variants near the end of the gene, all *CEP55* truncating variants reported in other relevant studies (Bondeson et al., 2017; Rawlins et al., 2019; Sparks et al., 2020), including ours, were located upstream or within the TSG101 and ALIX binding domain, suggesting a failed recruitment of ESCRT machinery during cytokinesis. Therefore, the pathomechanisms of upstream and downstream truncating variants in the *CEP55* gene were likely to be different. Moreover, qPCR on heterozygous parents in previous studies revealed almost equal levels of wild-type and truncated transcript, regardless of upstream or downstream truncation, indicating that *CEP55* truncating variants were not targeted by nonsense-mediated mRNA decay pathway (Bondeson et al., 2017; Frosk et al., 2017).

As shown in Table 1, all *CEP55*-associated dead fetuses harbor biallelic truncating variants except one case with a truncating mutation and a non-truncating (missense) mutation c.1373A > G (p.His458Arg) (Sparks et al., 2020). The exceptional case was described to have hydrocephaly, renal dysplasia, skin edema, clubfoot, oligohydramnios, and complex congenital heart disease, which was consistent with the phenotype of fetus II:3 in our study (Sparks et al., 2020). In view that the missense mutation c.1373A > G (p.His458Arg) is located within the last 40 amino acids, a region essential for localisation to the midbody during cytokinesis (Frosk et al., 2017), we speculate that it might also disrupt localisation of CEP55 to the midbody of dividing cells.

Furthermore, Barrie et al. (2020) depicted a viable phenotype spectrum of *CEP55*-associated disease in seven cases, all of whom survived the perinatal period and displayed microcephaly, intellectual disability, and mild skeletal abnormalities without renal involvements. Of these patients, four carried compound heterozygous *CEP55* variants comprised of a common missense mutation c.70G > A p.(Glu24Lys) and a nonsense mutation, while the other three siblings had a homozygous downstream splice site variant c.1191 + 2T > C. The effect of non-truncating variants on *CEP55* function remains unclear.

It was previously established that depletion of CEP55 caused abscission failure in most human cells (such as HeLa cells) *in vitro* (Fabbro et al., 2005; Lee et al., 2008; van der Horst et al., 2009). However, recent *in vivo* evidence from *CEP55*-knockout mice has shown that CEP55 is dispensable for abscission in most cell types except neural stem cells (NSCs), explaining binucleation only in neurons but not other tissues in dead fetuses with *CEP55* variants. Even in NSCs where CEP55 and ESCRT are required for survival and abscission, CEP55 just ensures the speed and success of abscission but is not absolutely necessary (Tedeschi et al., 2020; Little and Dwyer, 2021; Little et al., 2021). Further *in vivo* experiments are needed to elucidate an alternative CEP55-independent pathway for midbody ESCRT recruitment or even an ESCRT-independent cell division mechanism. Additionally, CEP55 has been proposed to function as a key regulator of cilia disassembly through stabilizing Aurora A kinase (Zhang et al., 2021).

Our case report elaborately presents the phenotypes of two Chinese stillborn babies linked to *CEP55* variants. This is the first report of *CEP55*-associated lethal fetal syndrome in China, and endocardial cushion defects observed in fetus II:3 is firstly described in such patients. However, there are some limitations in our study: 1) as mentioned above, WES was not performed on fetus II:3 owing to the unavailability of samples; 2) autopsy was not conducted due to autolysis; 3) functional investigations were absent.

## Conclusion

We describe a Chinese family with multiple stillbirths due to biallelic nonsense *CEP55* variants. Our findings extend and summarize the genotypic and phenotypic features of *CEP55*-associated lethal fetal syndrome, equipping clinicians with crucial insights when encountering a family with stillbirths displaying complex brain and kidney malformations.

## Data Availability

The datasets for this article are not publicly available due to concerns regarding participant/patient anonymity. Requests to access the datasets should be directed to the corresponding authors.
